# False negative assessments: an effective quality assurance method

**DOI:** 10.1186/bcr3300

**Published:** 2012-11-09

**Authors:** DD Manuel, BJG Dall, N Sharma

**Affiliations:** 1 Leeds Teaching Hospital NHS Trust, Leeds, UK

## Objective

To identify the frequency and characteristics of false negative assessment (FNA); an interval cancer with prior recall to assessment.

## Methods

A retrospective audit, over 7 years, between 1 April 2004 and 31 March 2011. Using NBSS databases, we recorded: lesion type, size, further mammographic views, ultrasound, clinical findings, biopsy results and final histology.

## Results

Twenty-nine cases by QARC, 13 true FNA and 16 excluded because contralateral or in a different quadrant. In this period, 220,522 woman were screened and 10,391 (<5%) were referred for assessment. Total cancers detected in this period were 2,343, of which 1,867 were diagnosed at assessment and 476 (20%) were interval cancers. True FNA: 2.7% of all interval cancers. True FNA: 0.6% of all screen-detected cancers. All cases were background mixed density. True FNA: 11/13 incident screen, 2/13 prevalent. Asymmetry/stromal deformity in 7/13 (54%), calcification 4/13 (30%), mass 2/13 (15%). Two cases were early recall (EC) (asymmetry, and mass with calcifications), both incident screens. EC did not contribute in either case as it led to false reassurance. All asymmetry/stromal deformity reassuring on further views, with no correlating ultrasound or clinical abnormality. On review of all cases, only 2/13 were felt not to have had complete triple assessment. Final pathology: two DCIS, four lobular cancers, seven ductal cancers. Seven cases were discussed at a multidisciplinary meeting.

## Conclusion

True FNA remains very low. This is because assessment cases have complete triple assessment and MDT discussion. FNA can be used as a valuable educational process and mechanism to ensure consistency and adherence to NHSBSP standards.

